# As(V) and P Competitive Sorption on Soils, By-Products and Waste Materials

**DOI:** 10.3390/ijerph121215016

**Published:** 2015-12-10

**Authors:** Ivana María Rivas-Pérez, Remigio Paradelo-Núñez, Juan Carlos Nóvoa-Muñoz, Manuel Arias-Estévez, María José Fernández-Sanjurjo, Esperanza Álvarez-Rodríguez, Avelino Núñez-Delgado

**Affiliations:** 1 Department of Soil Science and Agricultural Chemistry, Engineering Polytechnic School, University of Santiago de Compostela, Lugo 27002, Spain; ivanamaria.rivas@rai.usc.es (I.M.R.-P.); mf.sanjurjo@usc.es (M.J.F.-S.); esperanza.alvarez@usc.es (E.A.-R.); 2 Department of Plant Biology and Soil Science, Faculty of Sciences, Campus Ourense, University of Vigo, Ourense 32004, Spain; remigio.paradelo@uvigo.es (R.P.-N.); edjuanca@uvigo.es (J.C.N.-M.); mastevez@uvigo.es (M.A.-E.)

**Keywords:** arsenic, forest soil, phosphorus, sorption-competition, vineyard soil, waste materials

## Abstract

Batch-type experiments were used to study competitive As(V) and P sorption on various soils and sorbent materials. The materials assayed were a forest soil, a vineyard soil, pyritic material, granitic material, coarsely and finely ground mussel shell, calcinated mussel shell ash, pine sawdust and slate processing fines. Competition between As(V) and P was pronounced in the case of both soils, granitic material, slate fines, both shells and pine sawdust, showing more affinity for P. Contrary, the pyritic material and mussel shell ash showed high and similar affinity for As(V) and P. These results could be useful to make a correct use of the soils and materials assayed when focusing on As and P removal in solid or liquid media, in circumstances where both pollutants may compete for sorption sites.

## 1. Introduction

Arsenic pollution is a matter of public health concern, mainly in connection with its presence in drinking water. The use of wood preservative compounds that include arsenic can originate arsenic pollution episodes in forest areas [1], and As-based herbicides could have similar effect when used in vineyard soils [2], thus increasing overall risks of soil and water pollution [3]. In addition, diverse anthropogenic sources, such as agriculture, mining and industrial activities, can cause environmental P pollution, which can lead to eutrophication [4,5].

In some cases, soils can suffer the simultaneous application of P (*i.e.*, by fertilization) and As (*i.e.*, by spreading of pesticides), causing potential competition for adsorption sites, which could be very relevant [6,7,8]. In this regard, enhanced risks of soil and water pollution could take place if phosphate competition inhibits arsenic sorption and/or causes arsenic release from previously occupied sorption sites.

Previous publications have studied As retention/release on soils and various sorbent materials [9,10,11], and other studies have focused on P retention/release [12,13,14,15,16]. Recent works have also evaluated different sorbent materials as regards the influence of the simultaneous presence of two or more anions -including arsenate and phosphate- on its respective sorption results [17,18,19,20].

However, phosphate/arsenate competitive sorption has not yet been studied in many soils. In addition, the simultaneous phosphate/arsenate retention potential, as well as characteristics corresponding to competition for sorption sites, are not yet known for many sorbent materials.

In view of that, in this work we study As(V) and P competitive sorption on different soils and materials, concretely a forest soil, a vineyard soil, a pyritic material, a granitic material (all of them being soils and/or degraded environments that could receive the simultaneous application of P fertilization and As-based pesticides), coarse and fine mussel shell, calcinated mussel shell ash, pine sawdust, slate processing fines (all of these materials being by-products that could act as bio-sorbents, especially interesting if having the potential of efficiently removing phosphate and arsenate simultaneously). The results could be used to program appropriate management practices for these soils, as well as the correct recycling of the studied by-products as bio-sorbents, using them when justified in solid or liquid media where both pollutants are present simultaneously.

## 2. Material and Methods

### 2.1. Materials

The materials used were a forest soil, a vineyard soil, pyritic material, granitic material, finely (<1 mm) and coarsely (0.5–3 mm) ground mussel shell, calcinated mussel shell ash, pine sawdust and slate processing fines. Most of those materials were previously described by Seco-Reigosa *et al.* [21]. Additionally, Osorio-López *et al.* [22] described the vineyard soil, Seco-Reigosa *et al.* [23] described the mussel shell ashes, Otero *et al.* [24] described the pyritic material and the forest soil, and, finally, Seco-Reigosa *et al.* [25] described the granitic material. In these previous works we studied sorption kinetics, as well as the fitting to isotherm adsorption models, the effects of factors such as concentration, pH, and some competitive anions, and also fractionation and the effect of incubation time on sorption of As(V), Cr(VI) and/or Hg(II). However, none of these previous works have studied P and As(V) competition for sorption sites, which is the aim of the present work.

The forest soil samples corresponded to an A horizon in a soil developed over granitic rocks in the vicinity of the Alcoa aluminum factory (San Cibrao, Lugo Province, Spain), with dominance of *Eucalyptus globulus* as tree species. The vineyard soil was sampled in Sober (Lugo Province, Spain). The pyritic material was from a copper mine spoil (Touro, A Coruña Province, Spain). The granitic material was from Santa Cristina (Ribadavia, Ourense Province, Spain), and was similar to a C horizon derived from a rocky substrate, nowadays exposed to the atmosphere after the elimination of the upper horizons. The finely and coarsely crushed mussel shells were from the Abonomar S.L. factory (A Illa de Arousa, Pontevedra Province, Spain). Mussel shell ash was from Calizamar S.L. (Boiro, A Coruña Province, Spain). Pine-sawdust was a commercial product from Vitakraft (Las Rozas, Spain), sold in the market. The slate processing fines were from the slate-processing enterprise Europizarras S.L. (A Fonsagrada, Lugo Province, Spain).

The forest and vineyard soils, as well as the pyritic and granitic materials, were sampled in a zigzag manner (0–20 cm depth), with 10 subsamples taken to perform each final composite sample. These samples were transported to the laboratory to be air dried and sieved through 2 mm. Finally, chemical determinations were carried out on the <2 mm fraction. Determinations were performed in triplicate on all materials.

**Table 1:** General characteristics of the soils and the sorbent materials (average values for 3 replicates, with coefficients of variation always <5%).

Parameter	Forest Soil	Vineyard Soil	Pyritic Material	Granitic Material	Coarse Shell	Fine Shell	Shell Ash	Pine-Sawdust	Slate Fines
C (%)	4.22	2.94	0.26	0.11	12.67	11.43	13.21	46.13	0.2
N (%)	0.33	0.23	0.04	0.04	0.36	0.21	1.13	0.03	0.02
pH_water_	5.65	4.48	2.97	5.72	9.11	9.39	12.54	4.91	8.61
Ca_e_ (cmol·kg^−1^)	4.37	1.78	0.36	0.18	12.64	24.75	39.27	5.39	4.31
Mg_e_ (cmol·kg^−1^)	0.66	0.24	0.29	0.13	0.58	0.72	7.47	1.37	0.31
Na_e_ (cmol·kg^−1^)	0.33	0.14	0.14	0.27	5.24	4.37	19.92	0.66	0.63
K_e_ (cmol·kg^−1^)	0.60	0.83	0.24	0.31	0.31	0.38	2.61	1.55	0.31
Al_e_ (cmol·kg^−1^)	1.92	2.28	2.86	1.63	0.04	0.03	0	0.05	0.01
CIC_e_ (cmol·kg^−1^)	7.88	5.27	3.89	2.53	18.82	30.25	69.28	9.02	5.57
P_Olsen_ (mg·kg^−1^)	28.80	147.64	8.80	2.56	23.21	54.17	534.6	11.47	0.93
P_T_ (mg·kg^−1^)	423.9	679.3	606.3	88.62	186.5	101.5	1617	88.04	661.3
Ca_T_ (mg·kg^−1^)	708.5	607.1	603	<0.01	298,085	280,168	247,859	8088	2810
Mg_T_ (mg·kg^−1^)	830.5	5003	8384	355	1020	980.6	5286	164.4	11,797
Na_T_ (mg·kg^−1^)	515.1	297.6	412	102	5508	5173	8074	98.35	53.72
K_T_ (mg·kg^−1^)	1544	5441	3186	1434	80.57	202.1	896	540.7	991.3
As_T_ (mg·kg^−1^)	4.18	3.41	7	3	0.48	1.12	1.71	0.39	3.1
Cd_T_ (mg·kg^−1^)	0.43	0.14	0.08	<0.001	0.02	0.07	63.09	50.82	95.18
Cr_T_ (mg·kg^−1^)	18.35	41.44	99	3	1.32	4.51	4596	234.2	54,010
Cu_T_ (mg·kg^−1^)	15.72	521.1	773	7	3.20	6.72	31.75	14.87	30.95
Ni_T_ (mg·kg^−1^)	10.69	21.73	5	1	5.64	8.16	3421	260.6	24,737
Zn_T_ (mg·kg^−1^)	36.74	49.57	58	18	7.71	7.66	18.75	0	36.89
Mn_T_ (mg·kg^−1^)	92.99	305.4	296	24	5.70	33.75	18.67	5.19	28.46
Al_T_ (mg·kg^−1^)	22,676	25,664	9624	5981	93.89	433.2	3421	260.7	24,737
Fe_T_ (mg·kg^−1^)	9486	21,284	135,157	3505	170.37	1855	4596	234.2	54,010
Al_o_ (mg·kg^−1^)	4275	2003	563	1425	85.00	178.3	1733	112.5	730.6
Fe_o_ (mg·kg^−1^)	2333	1239	41,860	224	42.67	171.0	1659	15.62	1256

X_e:_ exchangeable concentration of the element; X_T_: total concentration of the element; Al_o_, Fe_o_: extracted with ammonium oxalate.

### 2.2. Methods

#### 2.2.1. Characterization of the Materials Used

C and N were measured on 5 g samples using an elemental Tru Spec CHNS auto-analyzer (LECO Corporation, St. Joseph, MI USA) [26]. A pH-meter (model 2001, Crison, L’Hospitalet de Llobregat, Barcelona, Spain) was used to measure pH in water (10 g of solid sample, with solid: liquid relation 1:2.5) [27]. A 1 M solution NH_4_Cl was used on 5 g samples to displace the exchangeable cations, then Ca, Mg and Al were quantified by atomic absorption spectroscopy, and Na and K by atomic emission spectroscopy (AAnalyst 200, Perkin Elmer, Boston, MA, USA) [28]; the effective cationic exchange capacity (eCEC) was calculated as the sum of all these cations [29]. Available P was determined as per Olsen and Sommers [30] using 5 g samples. Total concentration of P was determined by means of UV-visible spectroscopy (UV-1201, Shimadzu, Kioto, Japan) after nitric acid (65%) microwave assisted digestion on 1 g samples [31]. Total concentrations of Na, K, Ca, Mg, Al, Fe, Mn, as well as As, Cd, Cr, Cu, Ni and Zn, were determined using ICP-mass spectrometry (820-NS, Varian, Palo Alto, CA, USA), after nitric acid (65%) microwave assisted digestion on 1 g samples [32]. Ammonium oxalate solutions were used to obtain total non-crystalline Al and Fe (Al_o_, Fe_o_) from 1 g samples [33]. All trials were performed by triplicate. Table 1 shows the results corresponding to the chemical characterization of the materials assayed.

#### 2.2.2. As(V) and P Competitive Sorption

Triplicate samples of each material were added with the same P concentration (3 mmol·L^−1^) in all cases, and, simultaneously, different As(V) concentrations (0, 0.5, 1.5, 3 and 6 mmol·L^−1^) were also added. In parallel, using other triplicate samples of the materials, each one was added with the same As(V) concentration in all cases (3 mmol·L^−1^), and, simultaneously, different P concentrations (0, 0.5, 1.5, 3 and 6 mmol·L^−1^) were also added.

In all cases, 3 g of each solid sample (<2 mm fraction) were added with 30 mL of NaNO_3_ 0.01 M dissolutions containing the concentrations of As(V) and P indicated above. The resulting suspensions were shaken for 24 h, centrifuged at 4000 rpm (6167× *g*) for 15 min, and finally filtered using acid-washed paper. In the equilibrium dissolutions, P was determined by means of UV-visible spectroscopy (UV-1201, Shimadzu) [31], and As was quantified by means of ICP-mass spectrometry (820-NS, Varian). Sorbed As and P were calculated as the difference between added As and P, and As and P remaining in the equilibrium solution. As and P were determined by triplicate in all samples.

## 3. Results

### 3.1. Sorbed As When a Constant P Concentration and Increasing As Concentrations Are Added, and Sorbed P When a Constant As Concentration and Increasing P Concentrations Are Added

**Figure 1:**
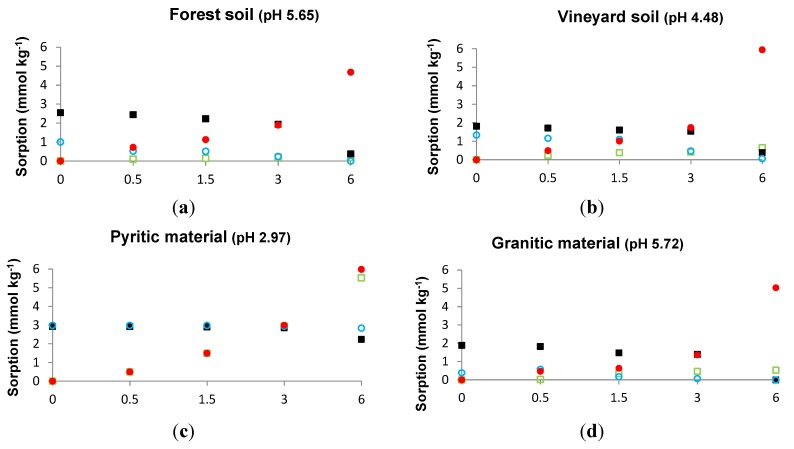

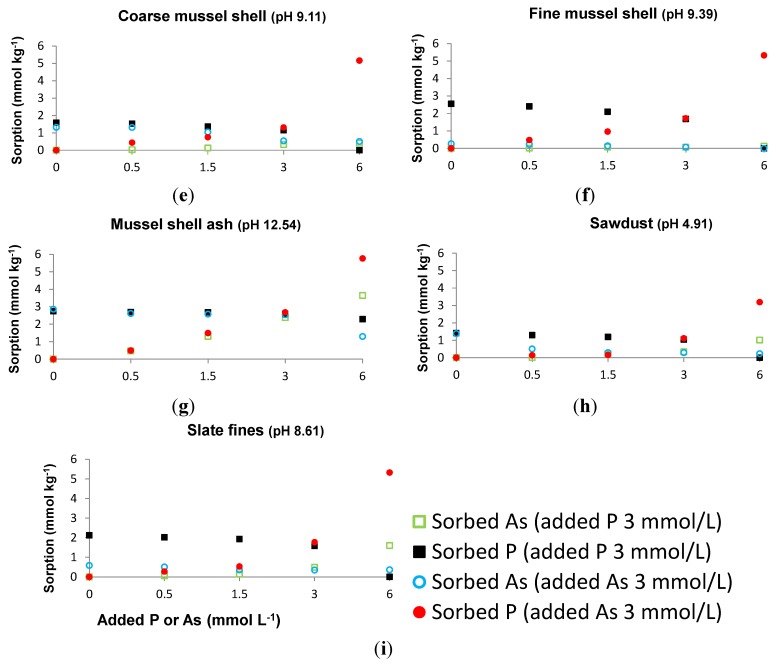
Sorbed As (empty green squares) and sorbed P (filled black squares) when added P is 3 mmol·L^−1^ and added As is increased from 0 to 6 mmol·L^−1^. Sorbed As (empty blue circles) and sorbed P (filled red circles) when added As is 3 mmol·L^−1^ and added P is increased from 0 to 6 mmol·L^−1^. (**a**) forest soil; (**b**) vineyard soil; (**c**) pyritic material; (**d**) granitic material; (**e**) coarse shell; (**f**) fine shell; (**g**) shell ash; (**h**) pine-sawdust; (**i**) slate fines.

Figure 1 shows sorbed As (or sorbed P) when a constant P (or As) concentration (3 mmol·L^−1^) and increasing As (or P) concentrations (0 to 6 mmol·L^−1^) are added to the various soils and sorbent materials. The results indicate that sorption was higher for P than for As in the case of a group of materials: both soils, granitic material, fine and coarse mussel shell, sawdust and slate fines.

Similarly, As sorption progressively decreased as a function of the increasing P concentration added, and, again, sorption was higher for P than for As when the added concentration was coincident for both elements (3 mmol·L^−1^). These results indicate that As and P competed for sorption sites in that group of materials, and that affinity was higher for P than for As. Another group of materials was constituted by the pyritic material and mussel shell ash, differing from the previous group in the fact that both sorbents exhibited similar affinity for As and P.

### 3.2. As and P Sorbed in the Absence or in the Presence of Each Other

**Figure 2:**
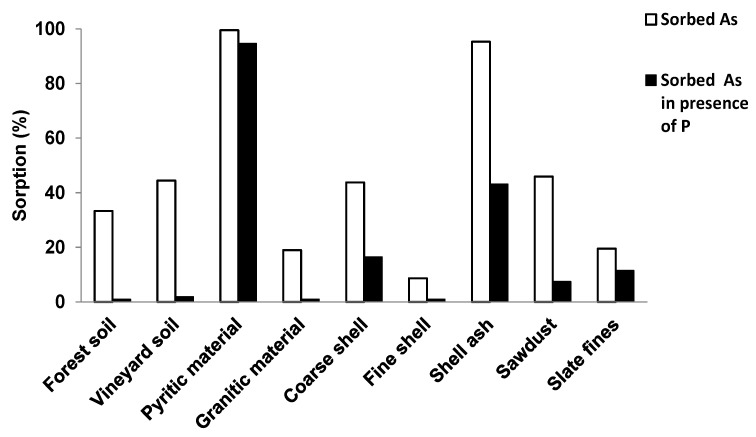
Sorbed As (%) when a concentration of 3 mmol As(V) L^−1^ is added, showing results in absence of P and in presence of 6 mmol P L^−1^.

Figure 2 shows percentage As sorbed in absence of P and in presence of 6 mmol P L^−1^, with 3 mmol As(V) L^−1^ added to the various soils and sorbent materials in both cases.

When 6 mmol·P·L^−1^ were added, the first group of materials previously commented (both soils, granitic material, both kinds of mussel shell, sawdust and slate fines) suffered a marked decrease in As sorption, up to near-zero values, making evident the importance of competition between both elements. However, P addition did not cause a relevant effect on As sorption on the pyritic material, whereas a more pronounced decrease in As sorption was detected in the case of mussel shell ash. Any case, As sorption was clearly higher on the pyritic material and mussel shell ash, as compared to the first group of materials. 

**Figure 3:**
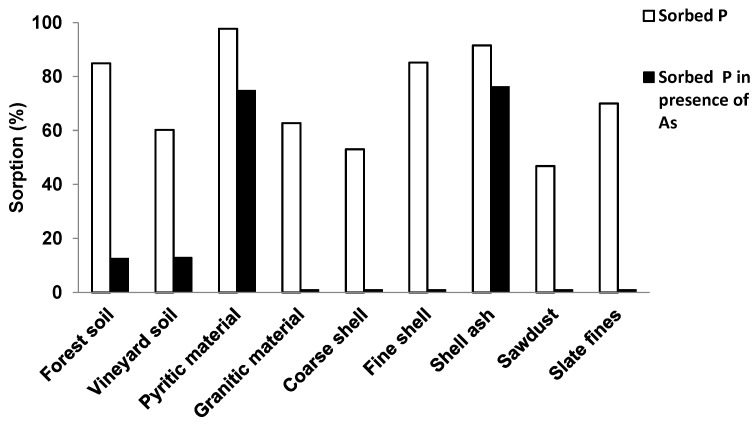
Sorbed P (%) when a concentration of 3 mmol P L^−1^ is added, showing results in absence of As and in presence of 6 mmol As L^−1^.

Figure 3 shows percentage P sorbed, comparing results in absence of As and in presence of 6 mmol As L^−1^, with a concentration of 3 mmol P L^−1^ being added to the various soils and sorbent materials in all cases. When 6 mmol As L^−1^ were added, P sorption decreased 80% in the case of fine mussel shell, and more than 50% in coarse mussel shell, both soils, granitic material and slate fines, whereas the decrease was not higher than 20% in the pyritic material and mussel shell ash.

## 4. Discussion

### 4.1. As and P Competition

Previous studies found the existence of competitive interactions between arsenate and phosphate [34,35], which were justified by the fact that both anions have similar chemical characteristics and affinity for protons (pK_a_ values 7.0 and 7.2 for arsenate and phosphate, respectively) [36]. Concretely, both anions have high chemical affinity for Fe and Al oxides [37,38,39,40], and for clay minerals [41].

Regarding competitive trials, Violante and Pigna [42] found lower sorption for AsO_4_^3−^ than for PO_4_^3−^ in organic-matter-rich soils, kaolinite, halloysite and non-crystalline Al minerals, thus being coincident with the results corresponding to some of our materials: both soils and the granitic material. Smith *et al.* [43] found that the presence of 0.16 mmol P L^−1^ greatly decreased As(V) sorption by soils containing low amounts of Fe oxides, indicating competition for sorption sites, but the presence of a similar amount of P had little effect on As(V) adsorbed by soils with high Fe content, although As(V) sorption substantially decreased when P concentration was increased to 3.2 mmol·L^−1^ in selected soils.

Sø *et al.* [35] showed that arsenate sorption on calcite was clearly decreased in presence of phosphate, whereas phosphate sorption was just slightly decreased by the presence of arsenate, which is coincident with the results of just one of our materials: mussel shell (a calcite-rich material). This behavior can be due to a better fitting for PO_4_^3−^ than for AsO_4_^3−^ on the surface of calcite [44,45,46].

It is especially relevant the fact that our pyritic material (rich in Fe oxides) and our mussel shell ash showed high sorption potential for both anions, with very low competitive effect between them. Hongshao and Stanforth [37] found similar results using goethite, and considered that the cause was that both anions have similar binding energy on that material. Thus, the fact that our pyritic material and shell ash have high affinity for As and P could be of great importance, since these materials could be useful to treat polluted media where both contaminants are present, which was not previously postulated for these sorbents.

Regarding P retention, authors such as Buckingham *et al.* [15], Hinsinger [47], and Carreira *et al.* [48] consider that calcium carbonate and Fe and Al oxides play an important role on P sorption, and it is relevant that those chemical compounds are present in various materials assayed in this work. P sorption on the pyritic material can be due to its high Fe oxy-hydroxides (Fe_o_) content (Table 1), taking into account that these Fe oxy-hydroxides have positively charged surfaces at acid pH values, thus electrostatically binding to P anions. Another retention mechanism that can take place is precipitation, when phosphate interacts with Al from the exchange complex in the acid environment prevailing. Regarding P sorption on mussel shell ash, its alkaline pH (9.4) causes that the dominant P-species is HPO_4_^2−^ [49]; in addition, shell ash contains Ca, Fe, Na and K oxides and carbonates [21], facilitating precipitation of calcium carbonate, which can be transformed to more stable hydroxy-apatite [50]; another P-retention mechanism can be P sorption on the surface of calcium carbonate [51], and, finally, shell ash has high content of non-crystalline Fe and Al minerals (Table 1), negatively charged at alkaline pH values, which could facilitate phosphate sorption by cationic bridges, as previously found for As anions [21].

In the case of As, the high retention that took place on our pyritic material can be in relation with the dominance of H_2_AsO_4_^−^ as As(V) species in acid environments [52], which can be sorbed on non-crystalline minerals positively charged at that pH. Our mussel shell ash also showed high As retention potential, although its pH was alkaline (9.4), situation where the dominant As(V) species is HAsO_4_^2−^ [52], and where the non-crystalline minerals are not positively charged. But this kind of ash derive from calcination of mussel shell, process that causes partial transformations of carbonates to oxides, which have been associated to As sorption [53]. As previously noted, mussel shell ash also contains remarkable concentrations of Fe and Al non-crystalline minerals (Table 1), negatively charged at alkaline pH values, which can also facilitate As binding through cationic bridges.

It must be taken into account that the experiments here presented were conducted at fixed pH values for each individual soil or sorbent material. Bearing in mind that the number of available sorption sites is pH dependent, it is clear that the role of pH is not elucidated in this study, but it may be significant, as previously shown for these materials in the case of As(V), Cr(VI) and Hg(II) [21,22,23,24,25].

### 4.2. Implications of the Study

Competitive As(V) and P sorption results found for the soils and sorbent materials assayed can be used to correctly program their management and recycling. It can be especially relevant that the pyritic material and mussel shell ash studied have high affinity for As and P, making feasible its utilizations to treat polluted media where both contaminants are present.

## 5. Conclusions

We used batch-type experiments to investigate competitive adsorption of As(V) and P on two soils and various by-products and waste materials that were not previously characterized in this regard. Both soils, the granitic material, slate fines, fine and coarse mussel shell, and pine sawdust, showed marked As/P competition, and higher affinity for P, whereas affinity was similar for As and P in the case of the pyritic material and mussel shell ash, which showed high retention capacities. These results could be used in the design of treatment systems aimed to achieve As and P removal in polluted media where both contaminants are present, and are especially relevant and useful in the case of the pyritic material and mussel shell ash.
